# Differences in emotion recognition between nonimmersive versus immersive virtual reality: preliminary findings in schizophrenia and bipolar disorder

**DOI:** 10.1097/YIC.0000000000000576

**Published:** 2024-12-06

**Authors:** Mauro Scala, Daniel Sánchez-Reolid, Roberto Sánchez-Reolid, Patricia Fernández-Sotos, Verónica Romero-Ferreiro, Miguel Ángel Alvarez-Mon, Guillermo Lahera, Giuseppe Fanelli, Alessandro Serretti, Chiara Fabbri, Antonio Fernández-Caballero, Roberto Rodriguez-Jimenez

**Affiliations:** a Department of Biomedical and Neuromotor Sciences (DIBINEM), University of Bologna, Bologna, Italy; b Department of Psychiatry, Health Research Institute Hospital 12 de Octubre (imas12); c Department of Legal Medicine, Psychiatry and Pathology, Complutense University of Madrid (UCM); d Department of Psychology, Faculty of Biomedical and Health Sciences, European University of Madrid (UEM), Madrid; e Instituto de Investigación en Informática de Albacete, Universidad de Castilla-La Mancha (UCLM), Albacete; f CIBERSAM-ISCIII (Biomedical Research Networking Centre in Mental Health), Madrid; g Departamento de Sistemas Informáticos, Universidad de Castilla-La Mancha (UCLM), Albacete; h Department of Psychiatry and Mental Health of Cartagena, Cartagena; i Department of Medicine and Medical Specialities, University of Alcala, Alcala de Henares; j Department of Psychiatry and Mental Health, Infanta Leonor University Hospital; k Ramón y Cajal Institute of Sanitary Research (IRYCIS), Madrid, Spain; l Department of Human Genetics, Radboud University Medical Center, Donders Institute for Brain, Cognition and Behaviour, Nijmegen, The Netherlands; m Department of Medicine and Surgery, Kore University of Enna, Enna; n Oasi Research Institute-IRCCS, Troina, Italy

**Keywords:** dynamic virtual humans, emotion recognition, facial affect recognition, mania, psychosis, social cognition

## Abstract

Deficits in social cognition may impair emotional processing and facial emotional recognition (FER) in patients with bipolar disorder (BD) and schizophrenia. FER is generally explored using photographs or images of static faces that do not fully capture the complexity of real-life facial stimuli. To overcome this limitation, we developed a set of dynamic virtual faces depicting six basic emotions (i.e. happiness, sadness, anger, fear, disgust, and surprise) and a neutral expression suitable for presentation in immersive and nonimmersive virtual realities. This study presents preliminary findings on the differences in FER accuracy from a frontal view between immersive and nonimmersive virtual realities among patients experiencing a relapse of schizophrenia (*n* = 10), a manic phase of BD (*n* = 10), and a group of healthy controls (HCs) (*n* = 10). As a secondary objective, we compare the FER accuracy across these three groups. Patients with schizophrenia and BD showed similar accuracy in recognizing emotions in immersive and nonimmersive virtual reality settings. However, patients with schizophrenia exhibited lower FER accuracy than HCs in both settings. Individuals with BD showed intermediate accuracy between those with schizophrenia and HCs, although these differences were not statistically significant. Notably, recognition of negative emotions was significantly impaired in both groups of patients.

## Introduction

Schizophrenia and bipolar disorder (BD) are chronic and severe mental disorders associated with significant levels of disability, with prevalence rates of 0.6–1% and 3–5%, respectively (Senner *et al*., 2023). Neuro and social cognitive deficits are commonly described in both disorders and lead to significant impairment in clinical and functional recovery (Harvey *et al*., 2022).

Social cognition refers to the mental processes involved in recognizing, perceiving, and understanding social information, thereby facilitating interpersonal interactions (Frith and Frith, 2007). Theory of mind, social perception, attributional style, and emotional processing are the four main domains of social cognition (Pinkham *et al*., 2014, 2016). Emotional processing, defined as recognizing, facilitating, and regulating emotions, relies on facial emotional recognition (FER), which refers to the ability to discern emotional states through facial expressions (Pinkham *et al*., 2014).

Patients with schizophrenia and BD share deficits in FER and typically perceive faces as a combination of separate parts rather than as whole entities (De Prisco *et al*., 2023; Miola *et al*., 2023). Notably, individuals with schizophrenia show more severe impairments in FER compared to patients with BD (De Prisco *et al*., 2023).

Different methodologies exist for exploring the FER. Previous studies have used stimuli such as photographs, static images, or videos of faces, which do not fully capture the richness of facial expressions (Davis and Gibson, 2000; Collignon *et al*., 2008). These tools have significant limitations in terms of validation, scene duration, and format (Edwards *et al*., 2002). As a result, virtual reality technologies have emerged as highly promising tools for simulating emotion recognition in real-life scenarios, providing dynamic avatars that replicate facial expressions in both nonimmersive (via a computer monitor) and immersive (via fully immersive vision glasses) settings (Fernández-Caballero *et al*., 2017).

In line with this, our group developed a set of dynamic virtual faces (DVFs) representing the six basic emotions (i.e. happiness, sadness, anger, fear, disgust, and surprise) and a neutral expression. These DVFs can be presented in immersive and nonimmersive virtual reality settings (García *et al*., 2020). Validation with 204 healthy controls (HCs), confirmed that the accuracy of FER with DVFs was at least comparable to standard tools such as the ‘Penn Emotion Recognition Test’ (ER-40) (Fernández-Sotos *et al*., 2021). Although previous studies have investigated nonimmersive FER in schizophrenia (Muros *et al*., 2021) and major depressive disorder (MDD) (Monferrer *et al*., 2023) using this set of DVFs, differences in FER accuracy between patients with schizophrenia and BD have not yet been explored.

The current study presents preliminary findings on the differences in FER accuracy between immersive and nonimmersive virtual reality settings among patients experiencing a relapse of schizophrenia, a manic phase of BD, and a group of HCs. Immersive virtual reality provides an environment with enhanced realism and ecological validity that closely mimics real-life social interactions, potentially improving engagement and motivation, which are critical for effective cognitive rehabilitation programs (Freeman *et al*., 2017). Although there is no comparative literature on differences in FER between immersive and nonimmersive virtual reality in patient groups, HCs demonstrated higher FER accuracy in immersive virtual reality (Vicente-Querol *et al*., 2023). The severe symptomatology experienced during the acute phases exacerbates impairments in social cognition (Lembke and Ketter, 2002; Li *et al*., 2010). Hence, identifying FER deficits during these periods allows a better understanding of their state-dependent nature and is crucial for developing timely and targeted interventions. As a secondary objective, the study aims to provide a comparison of FER accuracy across these three groups. Thus, the following hypotheses were tested:

1. Hypothesis 1 (H1): In all groups, FER accuracy in the immersive virtual reality setting will be equal to or better than that in the nonimmersive virtual reality setting.

2. Hypothesis 2 (H2): Patients with schizophrenia will show lower FER accuracy than patients with BD and HCs. Participants with BD will demonstrate intermediate FER accuracy between schizophrenia and HCs groups.

## Materials and methods

### Study design

Participants were recruited between July 2023 and April 2024 at the Hospital Universitario 12 de Octubre, Madrid, Spain. The study was conducted according to the guidelines of the Declaration of Helsinki and was approved by the Clinical Research Ethics Committee of the Hospital Universitario 12 de Octubre (CEIm No.: 23/034; approved on 18 January 2023). Informed consent was obtained from all subjects involved in the study.

### Participants

This preliminary sample included 30 participants: 10 patients experiencing a clinical relapse of schizophrenia, 10 individuals with an acute manic episode of BD, and 10 HCs. A clinical assessment was conducted by senior psychiatrists, confirming that the patients met the Diagnostic and Statistical Manual of Mental Disorders, Fifth Edition, Text Revision (DSM-5-TR) criteria for schizophrenia relapses and acute manic episodes. The severity of the symptoms necessitated hospitalization in the psychiatric ward for intensive treatment. When the experimental procedure was performed, patients were already undergoing pharmacological treatment, which likely contributed to a reduction in the psychopathological scale scores. All participants were recruited from the same socio-cultural area and matched in terms of age, sex, and educational level. The diagnosis was made according to the Structured Clinical Interview for the DSM-5-TR.

### Experimental procedure

During a single session, 52 DVFs were presented to the participants in nonimmersive (via a computer monitor) and immersive (via fully immersive vision glasses) settings. The sequence of presentations alternated between the participants for nonimmersive and immersive virtual reality experiences. The software tool and the DVFs are publicly accessible at the Castilla-La Mancha University’s institutional repository, RUIdeRA (https://hdl.handle.net/10578/27021). The session duration ranged from 20 to 40 min and involved participants undergoing a brief tutorial before identifying facial emotions. Participants were required to label each expression they observed among seven alternatives: joy, sadness, anger, fear, disgust, surprise, or neutral. Each emotion was randomly displayed eight times, whereas the neutral expression was shown four times. The DVFs varied in dynamism, viewpoints (frontal, right lateral, and left lateral), ethnicity (Caucasian or African), age, and physical characteristics, including variations in eye color, skin tone, and hair. For this preliminary study, only frontal face presentations were considered, with a total of 26 DVFs in both the nonimmersive and immersive settings.

To test our hypotheses, we evaluated the FER accuracy by measuring the percentage of correctly identified facial expressions (De La Torre-Luque *et al*., 2022).

### Data analysis

Statistical analyses were conducted using IBM SPSS Statistics software (version 27). Descriptive statistics, including means, standard deviations, and percentages, were used to analyze the quantitative and qualitative variables, respectively. Given the non-normal distribution of the data and the small size of the samples, nonparametric methods were predominantly utilized to test the hypotheses, with statistical significance set at a *P* value <0.05, uncorrected because of the exploratory nature of the study. The Wilcoxon signed-rank test was applied to assess differences in performance within the same group of participants across both immersive and nonimmersive virtual realities. The Mann–Whitney test was used for comparisons between two groups, whereas the Kruskal–Wallis test was used to compare all three participant groups. Finally, the chi-square test was used to compare differences in qualitative variables.

## Results

Sociodemographic and clinical data of the participants are described in Table 1. Age, sex distribution, and years of education did not significantly differ among the three groups. A relapse of schizophrenia was indicated by a clinical presentation with a Positive and Negative Syndrome Scale mean score of 84.70 ± 11.73. A manic episode of BD was characterized by a clinical presentation with a Young Mania Rating Scale total score of 22.90 ± 4.20 and a Montgomery-Åsberg Depression Rating Scale total score of 4.1 ± 5.5.

**Table 1:** Sociodemographic and clinical data

	SCZ (*n* = 10)	BD (*n* = 10)	HCs (*n* = 10)	Statistics
Sociodemographic				
Gender (% female/male)	30/70%	60/40%	40/60%	χ²(2) = 1.900; *P* = 0.387
Age (years)	43.00 ± 13,69	52.70 ± 16,65	46.20 ± 15,37	H(2) = 2.422; *P* = 0.298
Education (years)	12.00 ± 2.67	11.00 ± 2.16	13.40 ± 2.50	H(2) = 4.346; *P* = 0.114
Clinical				
PANSS positive	21.60 ± 7.26	–	–	–
PANSS negative	23.40 ± 7.31	–	–	–
PANSS general psychopathology	39.70 ± 5.60	–	–	–
PANSS total score	84.70 ± 11.73	–	–	–
YMRS	–	22.90 ± 4.20	–	–
MADRS	–	4.1 ± 5.51	–	–
Lithium	0%	50%	–	–
Valproate	0%	30%	–	–
Mixed (lithium+ valproate)	0%	20%	–	–
FGA	0%	0%	-	-
SGA	90%	90%	-	-
Mixed (FGA+SGA)	0%	10%	–	–
Clozapine	30%	0%	–	–
CPZE (mg/day)	795.00 ± 538.91	720.00 ± 404.20	–	–

Data are expressed as mean ± SD or %.

BD, bipolar disorder; CPZE, chlorpromazine equivalent dose; FG, first-generation antipsychotics; HC, healthy control; MADRS, Montgomery-Asberg Depression Rating Scale; PANSS, Positive and Negative Syndrome Scale; SCZ, schizophrenia; SGA, second generation antipsychotics; YMRS, Young Mania Rating Scale.

### Comparison of facial emotional recognition accuracy between nonimmersive versus immersive virtual reality (H1)

The comparison of the FER accuracy scores between the nonimmersive and immersive virtual reality settings is detailed in Table 2.

**Table 2:** Differences in facial emotion recognition accuracy between immersive and nonimmersive virtual reality for schizophrenia, bipolar disorder, and healthy controls

Comparison among the three groups of immersive versus nonimmersive tasks^a^				
		Nonimmersive	Immersive	Statistics
HCs		81.54 ± 10.06%	88.85 ± 13.38%	Z = −1.994 *P* = 0.046
SCZ		53.08 ± 26.51%	54.62 ± 25.50%	Z = −0.060 *P* = 0.953
BD		59.62 ± 23.09%	57,69 ± 19.61%	Z = −0.656 *P* = 0.512

Differences in FER accuracy for both overall and individual emotions among the three groups^b^				
	HCs	SCZ	BD	Statistics
Nonimmersive				
Total emotions+ neutral expression	81.54 ± 10.06%	53.08 ± 26.51%	59.62 ± 23.09%	H(2) = 9.093, *P* = 0.011
Neutral	85.00 ± 33.75%	80.00 ± 34.96%	75.00 ± 35.36%	H(2) = 0.725, *P* = 0.696
Joy	92.50 ± 12.08%	57.50 ± 40.91%	65.00 ± 39.44%	H(2) = 5.038, *P* = 0.081
Sadness	67.50 ± 31.29%	37.50 ± 39.53%	50.00 ± 44.10%	H(2) = 2.504, *P* = 0.286
Anger	95.00 ± 10.54%	60.00 ± 33.75%	60.00 ± 35.75%	H(2) = 10.283, *P* = 0.006
Fear	77.50 ± 21.89%	25.00 ± 23.57%	42.50 ± 44.17%	H(2) = 7.898, *P* = 0.019
Disgust	62.50 ± 29.46%	50.00 ± 33.33%	55.00 ± 32.91%	H(2) = 1.028, *P* = 0.598
Surprise	92.50 ± 16.87%	75.00 ± 35.36%	77.50 ± 32.17%	H(2) = 2.002, *P* = 0.367
Immersive				
Total emotions+ neutral expression	88.85 ± 13.38%	54.62 ± 25.50%	57.69 ± 19.61%	H(2) = 12.766, *P* = 0.002
Neutral	75.00 ± 42.49%	70.00 ± 42.16%	85.00 ± 33.75%	H(2) = 0.870, *P* = 0.647
Joy	90.00 ± 24.15%	55.00 ± 45.34%	60.00 ± 31.62%	H(2) = 6.134, *P* = 0.047
Sadness	85.00 ± 21.08%	50.00 ± 28.87%	52.50 ± 43.22%	H(2) = 6.162, *P* = 0.046
Anger	92.50 ± 16.87%	57.50 ± 33.44%	62.50 ± 33.85%	H(2) = 7.862, *P* = 0.020
Fear	90.00 ± 17.48%	22.50 ± 29.93%	35.00 ± 37.64%	H(2) = 15.685, *P* < 0.001
Disgust	85.00 ± 21.08%	60.00 ± 35.75%	52.50 ± 34.26%	H(2) = 5.451, *P* = 0.066
Surprise	97.50 ± 7.91%	75.00 ± 37.27%	70.00 ± 30.73%	H(2) = 6.712, *P* = 0.035

Data are expressed as the mean percentage of correctly recognized facial emotions with standard deviations (mean ± SD).

*P*-values (*P* < 0.05) indicate significant differences in FER accuracy.

BD, bipolar disorder; HC, healthy controls; SCZ, schizophrenia.

a Done using the Wilcoxon signed-rank test.

b Done using the Kruskal–Wallis test.

Within the HCs group, we identified significant differences (*P* = 0.046) in overall emotion recognition, with higher FER accuracy in immersive virtual reality compared to nonimmersive reality. When considering individual emotions, this difference was significant for disgust recognition (*P* = 0.024), with higher accuracy in immersive virtual reality compared to nonimmersive reality. However, we did not observe differences in the BD and schizophrenia groups, neither in overall nor individual emotion recognition.

### Comparison of facial emotional recognition accuracy between schizophrenia, BD, and HCs (H2)

**Fig. 1:**
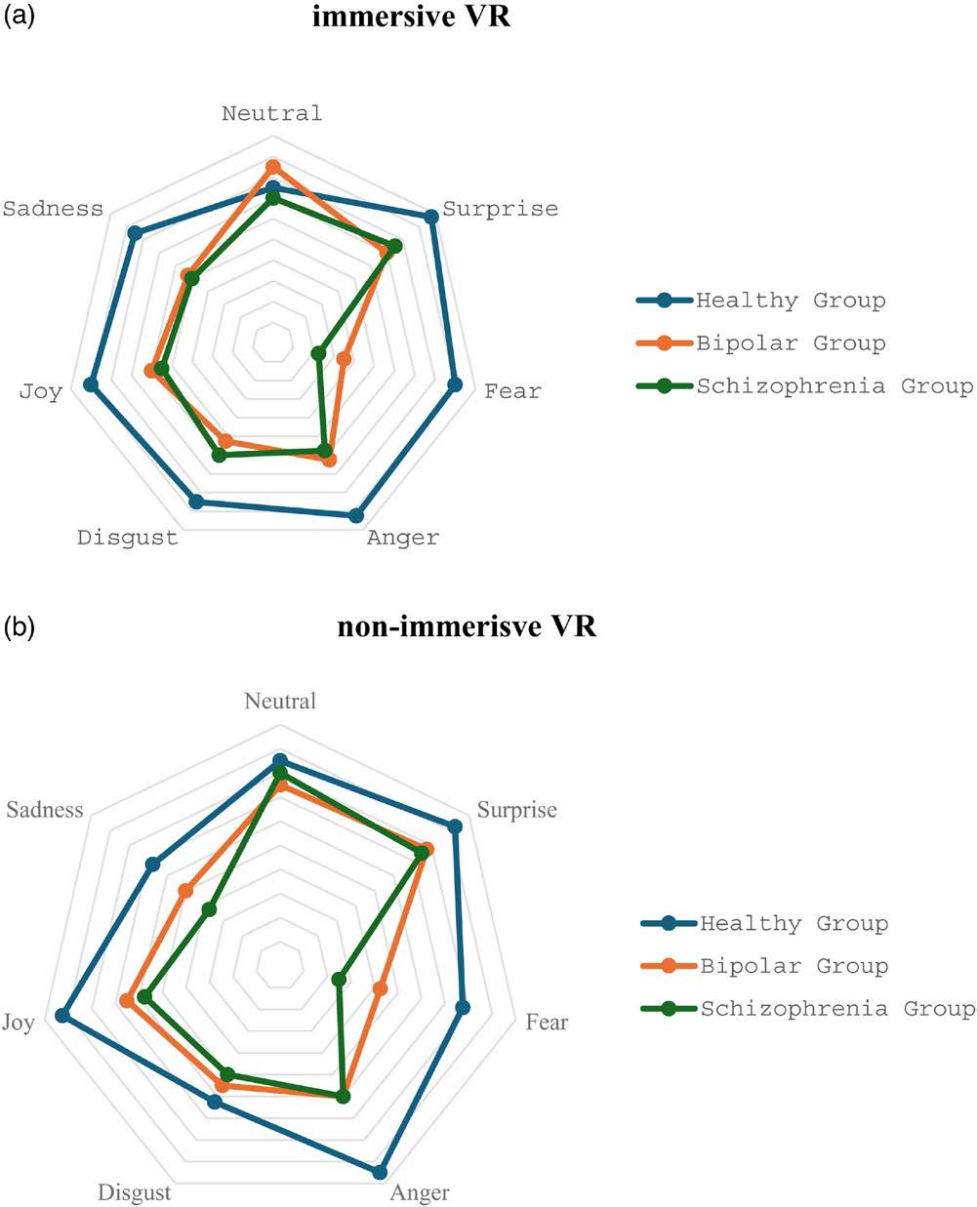
Graphical radar chart showing the percentage of facial emotions recognized by the schizophrenia, bipolar disorder, and healthy controls groups in (a) immersive and (b) nonimmersive virtual reality settings. Each line on the radar represents a percentage, with 10 lines ranging from 0% to 100%.

The FER accuracy scores among the three participant groups across both nonimmersive and immersive virtual reality experiences are detailed in Table 2 and visually depicted in Fig. 1.

### Nonimmersive virtual reality

A significant difference was observed in the overall emotion recognition scores among the three groups (*P* = 0.011). Post hoc tests specifically indicated differences between HCs and patients with schizophrenia (*P* = 0.015), with HCs showing a higher accuracy.

When analyzing each emotion individually, differences in FER emerged among the three groups for fear (*P* = 0.019) and anger (*P* = 0.006). Post hoc tests for fear demonstrated differences between the HCs and schizophrenia groups (*P* = 0.005), with the former showing higher accuracy. For anger, differences were observed between HCs and both the BD (*P* = 0.047) and schizophrenia groups (*P* = 0.043), with more accurate FER in HCs.

### Immersive virtual reality

Variability in overall emotion recognition between the three groups was consistent with the findings observed for nonimmersive virtual reality (*P* = 0.002), with post hoc analyses indicating significant differences between HCs and both the BD (*P* = 0.005) and schizophrenia groups (*P* = 0.007), with HCs demonstrating more accurate FER.

Regarding specific emotions, differences were observed for anger (*P* = 0.020), fear (*P* < 0.001), and surprise (*P* = 0.035). Post-hoc tests revealed that HCs showed more accurate recognition of anger compared to schizophrenia (*P* = 0.028), fear compared to BD (*P* = 0.008) and schizophrenia (*P* = 0.01), and surprise compared to BD (*P* = 0.031).

Although the post hoc tests did not reach statistical significance, the Kruskal–Wallis test indicated significant group differences in joy (*P* = 0.047) and sadness recognition (*P* = 0.046).

## Discussion

This study investigated FER in 10 patients experiencing a clinical relapse of schizophrenia and 10 individuals with an acute manic episode compared to 10 HCs. The assessment used both nonimmersive and immersive virtual reality settings with DVFs.

According to our first hypothesis, FER in immersive virtual reality was equal to or better than that in nonimmersive reality across all groups. Specifically, HCs showed better performance in immersive virtual reality, consistent with a previous study in healthy volunteers indicating their higher accuracy in this setting (Vicente-Querol *et al*., 2023). However, patients demonstrated equal FER accuracy between nonimmersive and immersive virtual reality. This could be attributed to the fact that the effective interpretation of DVFs in immersive virtual reality relies on intact neurocognitive function (David *et al*., 2014), and being clinically symptomatic may compromise a patient’s ability to fully benefit from the advantages of immersive virtual reality (Harvey *et al*., 2022). Therefore, virtual reality-based rehabilitation programs may provide greater benefits when used during the stable phases of the disease.

The second hypothesis of this study was only partially supported by our results. In terms of overall emotion recognition, patients with schizophrenia showed less accurate FER than HCs in both nonimmersive and immersive settings. Conversely, patients with BD showed decreased FER accuracy compared to HCs only in the immersive setting. The latter requires significant cognitive resources, which can be challenging for patients in manic episodes due to heightened distractibility, sensory sensitivity, and impaired attentional control (Fleck *et al*., 2009). These factors may lead to cognitive overload and difficulties in FER. Additionally, the elevated mood and impulsivity associated with mania can disrupt emotion processing and social cognition (Lembke and Ketter, 2002), making it harder to focus on subtle facial cues in immersive environments that simulate real-life interactions. Conversely, nonimmersive environments may place fewer demands on cognitive resources, enabling BD patients to perform similarly to HCs. Although individuals with BD demonstrated intermediate FER accuracy between patients with schizophrenia and HCs, these differences were not statistically significant for overall emotion recognition or for individual emotions. A larger sample size may be needed to reach adequate power to fully support our initial hypothesis.

Interestingly, in the immersive setting, patients with schizophrenia showed greater accuracy in recognizing disgust than those with BD, though this difference was not statistically significant. This may be due to the impaired ability to recognize disgust during manic episodes (Lembke and Ketter, 2002), with better recognition observed when patients with BD are euthymic (Harmer *et al*., 2002).

Differences between patients and HCs were particularly evident for some negative emotions, such as fear and anger, in both the nonimmersive and immersive virtual reality settings. This is consistent with previous studies using virtual dynamic stimuli in schizophrenia (Marcos-Pablos *et al*., 2016; Muros *et al*., 2021) and BD (Monferrer *et al*., 2024). Difficulties in recognizing fear and anger are evident from the earliest stages of schizophrenia (Tripoli *et al*., 2022), suggesting that these deficits could be distinct traits that persist throughout the disease. Additionally, impaired recognition of negative emotions during manic phases can be considered a mechanism that sustains the episode, known as the ‘mood congruity effect’, and is generally associated with the severity of manic symptoms (Turchi *et al*., 2016). Notably, individuals with BD underperformed HCs in the recognition of surprise. This may not be surprising, given that fear and surprise are generally considered to belong to the same emotional domain that occurs when facing an unexpected or uncertain stimulus (Jack *et al*., 2014; Gordillo *et al*., 2019).

These deficits in FER represent a significant limitation for the functional recovery of patients in their social, work, and family environments (Fett *et al*., 2011), and are not effectively addressed by current pharmacological approaches (Riccardi *et al*., 2021). Consequently, if confirmed in larger samples, these findings could have important implications for developing novel assessment and rehabilitation programs focused on social cognition and FER.

Despite being the first to investigate FER in two patient groups compared to HCs using both immersive and nonimmersive virtual realities, our results should be interpreted considering the limitations of this study. First, these preliminary findings were based on a small sample size, thus limiting their generalizability. Our sample size had a post hoc power of 0.80 to detect differences up to *d* = 1.2, therefore, smaller differences could not be detected (De Prisco *et al*., 2023). Furthermore, antipsychotic medication use may affect cognitive functioning (Haddad *et al*., 2023), which is crucial for FER (Ruihua *et al*., 2021). Considering the impaired attention and distractibility of patients in psychotic exacerbations or manic episodes, a 20–40-minute session may be overly long. Finally, although gender differences in FER were documented, particularly in schizophrenia (Weiss *et al*., 2007), the effect of gender on emotion perception was not considered in this preliminary study.

In summary, HCs showed higher accuracy in FER in immersive virtual reality compared to nonimmersive reality, whereas patients showed similar accuracy in both settings (H1). Regarding the differences between groups, although patients demonstrated lower accuracy in identifying emotions than HCs in both settings, there were no significant differences between schizophrenia and BD (H2). These conclusions are preliminary and should be interpreted cautiously because of the small sample size.

## Acknowledgements

The present study was conducted within a project approved by the Ethics Committee of Clinical Research (CEIm No.: 23/034; approved on January 18, 2023) of the ‘Hospital Universitario 12 de Octubre’, Madrid, Spain.

M.S., A.F.-C., D.S.-R., and R.R.-J. designed the study. M.S., P.F.-S., M.A.A.-M., A.S., V.R.-F., and G.L. managed the literature searches and analyses. M.S., D.S.-R., R.S.-R., A.S., G.F., and P.F.-S. selected the sample, evaluated patients and contributed in some aspects of the study design and in the interpretation of results. D.S.-R., V.R.-F. and R.R.-J. undertook the statistical analysis. M.S., D.S.-R., R.R.-J., C.F., G.F., and A.F.-C. wrote the first draft of the manuscript. All authors contributed to and have approved the final manuscript. Last two authors contributed equally to this work and share the last authorship.

### Conflicts of interest

The authors did not receive any grant from funding agencies in the public, commercial, or not-for-profit sectors. A.S. is or has been a consultant to or has received honoraria or grants unrelated to the present work from: Abbott, Abbvie, Angelini, Astra Zeneca, Clinical Data, Boheringer, Bristol Myers Squibb, Eli Lilly, GlaxoSmithKline, Innovapharma, Italfarmaco, Janssen, Lundbeck, Naurex, Pfizer, Polifarma, Sanofi, Servier, Taliaz. R.R.-J has been a consultant for, spoken in activities of, or received grants from: Instituto de Salud Carlos III, Fondo de Investigación Sanitaria (FIS), Centro de Investigación Biomédica en Red de Salud Mental (CIBERSAM), Madrid Regional Government (S2010/BMD-2422 AGES; S2017/BMD-3740; S2022/BMD-7216 AGES 3-CM), JanssenCilag, Lundbeck, Otsuka, Pfizer, Ferrer, Juste, Takeda, Exeltis, Casen-Recordati, Angelini, Rovi. For the remaining authors, there are no conflicts of interest.

## References

[R1] Collignon O Girard S Gosselin F Roy S Saint-Amour D Lassonde M Lepore F (2008). Audio-visual integration of emotion expression. Brain Res. 1242:126–135. 18495094 10.1016/j.brainres.2008.04.023

[R2] David DP Soeiro-de-Souza MG Moreno RA Bio DS (2014). Facial emotion recognition and its correlation with executive functions in bipolar I patients and healthy controls. J Affect Disord. 152-154:288–294. 24211178 10.1016/j.jad.2013.09.027

[R3] Davis PJ Gibson MG (2000). Recognition of posed and genuine facial expressions of emotion in paranoid and nonparanoid schizophrenia. J Abnorm Psychol. 109:445–450. 11016114

[R4] De La Torre-Luque A Viera-Campos A Bilderbeck AC Carreras MT Vivancos J Diaz-Caneja CM. (2022). Relationships between social withdrawal and facial emotion recognition in neuropsychiatric disorders. Prog Neuropsychopharmacol Biol Psychiatry. 113:110463. 34718073 10.1016/j.pnpbp.2021.110463

[R5] De Prisco M Oliva V Fico G Montejo L Possidente C Bracco L. (2023). Differences in facial emotion recognition between bipolar disorder and other clinical populations: a systematic review and meta-analysis. Prog Neuropsychopharmacol Biol Psychiatry. 127:110847. 37625644 10.1016/j.pnpbp.2023.110847

[R6] Edwards J Jackson HJ Pattison PE (2002). Emotion recognition via facial expression and affective prosody in schizophrenia. Clin Psychol Rev. 22:789–832. 12214327 10.1016/s0272-7358(02)00130-7

[R7] Fernández-Caballero A Navarro E Fernández-Sotos P González P Ricarte JJ Latorre JM Rodriguez-Jimenez R (2017). Human-avatar symbiosis for the treatment of auditory verbal hallucinations in schizophrenia through virtual/augmented reality and brain-computer interfaces. Front Neuroinform. 11:64. 29209193 10.3389/fninf.2017.00064 PMC5702358

[R8] Fernández-Sotos P García AS Vicente-Querol MA Lahera G Rodriguez-Jimenez R Fernández-Caballero A (2021). Validation of dynamic virtual faces for facial affect recognition. PLoS One. 16:e0246001. 33493234 10.1371/journal.pone.0246001 PMC7833130

[R9] Fett AKJ Viechtbauer W Dominguez MG Penn DL Van Os J Krabbendam L (2011). The relationship between neurocognition and social cognition with functional outcomes in schizophrenia: a meta-analysis. Neurosci Biobehav Rev. 35:573–588. 20620163 10.1016/j.neubiorev.2010.07.001

[R10] Fleck DE Shear PK Strakowski SM (2009). Manic distractibility and processing efficiency in bipolar disorder. The Neuropsychology of Mental Illness. Cambridge University Press; first ed., pp. 365–377.

[R11] Freeman D Reeve S Robinson A Ehlers A Clark D Spanlang B Slater M (2017). Virtual reality in the assessment, understanding, and treatment of mental health disorders. Psychol Med. 47:2393–2400. 28325167 10.1017/S003329171700040X PMC5964457

[R12] Frith CD Frith U (2007). Social cognition in humans. Curr Biol. 17:R724–R732. 17714666 10.1016/j.cub.2007.05.068

[R13] García AS Fernández-Sotos P Vicente-Querol MA Lahera G Rodriguez-Jimenez R Fernández-Caballero A (2020). Design of reliable virtual human facial expressions and validation by healthy people. Integr Comput-Aided Eng. 27:287–299.

[R14] Gordillo F Mestas L Pérez MA Escotto EA Arana JM (2019). The priming effect of a facial expression of surprise on the discrimination of a facial expression of fear. Curr Psychol. 38:1613–1621. 10.1017/sjp.2018.5 29490718

[R15] Haddad C Salameh P Sacre H Clément JP Calvet B (2023). Effects of antipsychotic and anticholinergic medications on cognition in chronic patients with schizophrenia. BMC Psychiatry. 23:61. 36694187 10.1186/s12888-023-04552-y PMC9872384

[R16] Harmer CJ Grayson L Goodwin GM (2002). Enhanced recognition of disgust in bipolar illness. Biol Psychiatry. 51:298–304. 11958780 10.1016/s0006-3223(01)01249-5

[R17] Harvey PD Bosia M Cavallaro R Howes OD Kahn RS Leucht S. (2022). Cognitive dysfunction in schizophrenia: an expert group paper on the current state of the art. Schizophr Res Cogn. 29:100249. 35345598 10.1016/j.scog.2022.100249 PMC8956816

[R18] Jack RE Garrod OGB Schyns PG (2014). Dynamic facial expressions of emotion transmit an evolving hierarchy of signals over time. Curr Biol. 24:187–192. 24388852 10.1016/j.cub.2013.11.064

[R19] Lembke A Ketter TA (2002). Impaired recognition of facial emotion in mania. Am J Psychiatry. 159:302–304. 11823275 10.1176/appi.ajp.159.2.302

[R20] Li H Chan RCK McAlonan GM Gong Q-yong (2010). Facial emotion processing in schizophrenia: a meta-analysis of functional neuroimaging data. Schizophr Bull. 36:1029–1039. 19336391 10.1093/schbul/sbn190 PMC2930350

[R21] Marcos-Pablos S González-Pablos E Martín-Lorenzo C Flores LA Gómez-García-Bermejo J Zalama E (2016). Virtual avatar for emotion recognition in patients with schizophrenia: a pilot study. Front Hum Neurosci. 10:421. 27616987 10.3389/fnhum.2016.00421 PMC4999437

[R22] Miola A Trevisan N Salvucci M Minerva M Valeggia S Manara R Sambataro F (2023). Network dysfunction of sadness facial expression processing and morphometry in euthymic bipolar disorder. Eur Arch Psychiatry Clin Neurosci. 274:525–536. 37498325 10.1007/s00406-023-01649-z PMC10995000

[R23] Monferrer M García AS Ricarte JJ Montes MJ Fernández-Caballero A Fernández-Sotos P (2023). Facial emotion recognition in patients with depression compared to healthy controls when using human avatars. Sci Rep. 13:6007. 37045889 10.1038/s41598-023-31277-5 PMC10097677

[R24] Monferrer M García AS Ricarte JJ Montes MJ Fernández-Caballero A Fernández-Sotos P (2024). Dynamic virtual faces demonstrate deterioration in the recognition of facial emotion in bipolar disorder patients. Curr Psychol. 43:15113–15124.

[R25] Muros NI García AS Forner C López-Arcas P Lahera G Rodriguez-Jimenez R. (2021). Facial affect recognition by patients with schizophrenia using human avatars. J Clin Med. 10:1904. 33924939 10.3390/jcm10091904 PMC8124197

[R26] Pinkham AE Penn DL Green MF Buck B Healey K Harvey PD (2014). The social cognition psychometric evaluation study: results of the expert survey and RAND panel. Schizophr Bull. 40:813–823. 23728248 10.1093/schbul/sbt081 PMC4059426

[R27] Pinkham AE Penn DL Green MF Harvey PD (2016). Social cognition psychometric evaluation: results of the initial psychometric study. Schizophr Bull. 42:494–504. 25943125 10.1093/schbul/sbv056 PMC4753585

[R28] Riccardi C Montemagni C Del Favero E Bellino S Brasso C Rocca P (2021). Pharmacological treatment for social cognition: current evidence. Int J Mol Sci. 22:7457. 34299076 10.3390/ijms22147457 PMC8307511

[R29] Ruihua M Hua G Meng Z Nan C Panqi L Sijia L. (2021). The relationship between facial expression and cognitive function in patients with depression. Front Psychol. 12:648346. 34234708 10.3389/fpsyg.2021.648346 PMC8256151

[R30] Senner F Hiendl L Bengesser S Adorjan K Anghelescu IG Baune BT. (2023). Medication adherence and cognitive performance in schizophrenia-spectrum and bipolar disorder: results from the PsyCourse Study. Transl Psychiatry. 13:99. 36966169 10.1038/s41398-023-02373-x PMC10039892

[R31] Tripoli G Quattrone D Ferraro L Gayer-Anderson C La Cascia C La Barbera D. (2022). Facial emotion recognition in psychosis and associations with polygenic risk for schizophrenia: findings from the multi-center EU-GEI case–control study. Schizophr Bull. 48:1104–1114. 35325253 10.1093/schbul/sbac022 PMC9434422

[R32] Turchi F Amodeo G Favaretto E Righini S Mellina E La Mela C Fagiolini A (2016). Le basi neurali della cognizione sociale nel disturbo bipolare [Neural basis of social cognition in bipolar disorder]. Riv Psichiatr. 51:177–189. 27869904 10.1708/2476.25886

[R33] Vicente-Querol MA Fernández-Caballero A González P González-Gualda LM Fernández-Sotos P Molina JP García AS (2023). Effect of action units, viewpoint and immersion on emotion recognition using dynamic virtual faces. Int J Neural Syst. 33:2350053. 37746831 10.1142/S0129065723500533

[R34] Weiss EM Kohler CG Brensinger CM Bilker WB Loughead J Delazer M Nolan KA (2007). Gender differences in facial emotion recognition in persons with chronic schizophrenia. Eur Psychiatry. 22:116–122. 17137757 10.1016/j.eurpsy.2006.05.003

